# Duration of Humoral and Cellular Immunity 8 Years After Administration of Reduced Doses of the 17DD-Yellow Fever Vaccine

**DOI:** 10.3389/fimmu.2019.01211

**Published:** 2019-06-21

**Authors:** Ismael Artur da Costa-Rocha, Ana Carolina Campi-Azevedo, Vanessa Peruhype-Magalhães, Jordana Grazziela Coelho-dos-Reis, Jordana Rodrigues Barbosa Fradico, Thalles Souza-Lopes, Laise Rodrigues Reis, Larissa Chaves Freire, Christiane Costa-Pereira, Juliana Vaz de Melo Mambrini, Maria de Lourdes de Sousa Maia, Sheila Maria Barbosa de Lima, Tatiana Guimarães de Noronha, Janaina Reis Xavier, Luiz Antonio Bastos Camacho, Elizabeth Maciel de Albuquerque, Roberto Henrique Guedes Farias, Thalita da Matta de Castro, Akira Homma, Alessandro Pecego Martins Romano, Carla Magda Domingues, Reinaldo de Menezes Martins, Andréa Teixeira-Carvalho, Olindo Assis Martins-Filho

**Affiliations:** ^1^Grupo Integrado de Pesquisas em Biomarcadores, Instituto René Rachou, Fundação Oswaldo Cruz – FIOCRUZ-Minas, Belo Horizonte, Brazil; ^2^Laboratório de Virologia Básica e Aplicada, Departamento de Microbiologia, Instituto de Ciências Biológicas, Universidade Federal de Minas Gerais, Belo Horizonte, Brazil; ^3^Núcleo de Estudos em Saúde Pública e Envelhecimento, Instituto René Rachou, Fundação Oswaldo Cruz – FIOCRUZ-Minas, Belo Horizonte, Brazil; ^4^Assessoria Clínica, Instituto de Tecnologia em Imunobiológicos Bio-Manguinhos – FIOCRUZ, Rio de Janeiro, Brazil; ^5^Laboratório de Tecnologia Virológica, Instituto de Tecnologia em Imunobiológicos Bio-Manguinhos – FIOCRUZ, Rio de Janeiro, Brazil; ^6^Departamento de Epidemiologia e Métodos Quantitativos em Saúde - Escola Nacional de Saúde Pública – FIOCRUZ, Rio de Janeiro, Brazil; ^7^Instituto de Biologia do Exército, Rio de Janeiro, Brazil; ^8^Departamento de Vigilância das Doenças Transmissíveis, Secretaria de Vigilância em Saúde, Ministério da Saúde, Brasília, Brazil

**Keywords:** Yellow Fever, 17DD vaccine, subdoses, neutralizing antibodies, cellular memory

## Abstract

The present study aims to determine whether 17DD-YF-specific humoral and cellular immunological memory is maintained 8-years after primary vaccination with subdoses (10,447IU;3,013IU;587IU;158IU;31IU). For this purpose, this follow-up study was carried out in a subset of volunteers (*n* = 98) originally enrolled in the dose-response study in 2009 and 46 non-vaccinated controls. Our results demonstrated that vaccinees, who had seroconverted following primary vaccination and had not been revaccinated, present similar neutralizing antibodies levels and YF-specific cellular memory, particularly CMCD4 and EMCD8 as compared to the reference full dose (27,476IU). Although, PRNT seropositivity rates were similar across subgroups (94, 82, 83, 94, 80, and 91%, correspondingly), only doses above 587IU elicited similar iterative proportion of seropositivity rates, calculated as a progressive decrease on seropositivity rates along time (89, 80, 80, and 91%, respectively) as compared to 158IU and 31IU (68 and 46%, respectively). Noteworthy were the strong positive correlations (“EMCD4,EMCD8” and “TNFCD8,IFNCD8”) observed in most subdoses, except for 31IU. Major similarities underscored the preserved antibody titers and the outstanding levels of EMCD8, relevant correlates of protection for YF-specific immunity. These findings provide evidences to support the regular use of dose sparing strategy for YF vaccine in adults.

## Introduction

Yellow fever (YF) is a severe acute febrile infectious disease, transmitted by mosquitoes infected with a Flavivirus RNA that occurs in Latin America and Africa. The disease is more frequent in non-immunized travelers entering into YF-endemic areas and non-vaccinated young males living in YF-endemic countries due to incursions in sylvatic areas of YF viral circulation (1, 2).

YF represents a substantial risk for non-immunized travelers entering YF-endemic areas and especially to residents of YF-endemic countries (1, 2). Because there is no effective treatment for YF, the prevention by immunization is critical to reduce the risks of YF infection. The YF vaccination has been considered the most relevant and effective prophylactic measure, inducing protective immunity within 10–30 days in ~95–99% of primary adult vaccinees (1, 3, 4).

The current outbreaks of YF in Brazil and Africa (5–10) have increased the demand for YF vaccine with consequent depletion of international stockpile. In response to this scenario, the World Health Organization (WHO) has recommended the use of fractional dose strategy to prevent the YF spread (11, 12). The fractional dose vaccination is indicated only during emergency response to YF outbreaks, when the shortage of YF-vaccine production or the need for the full-dose exceeds the capacity of the global stockpile. The WHO has developed an agenda to stimulate research to address policy-relevant issues. Critical questions still remain to be answered on the immunogenicity of fractional dose in young children (<2years), pregnant women, HIV-infected subjects as well as in immunocompromised patients. Moreover, a relevant issue is whether the immune responses to fractional dose are similar in populations with environmental exposures to other flaviviruses or flavivirus vaccination. Another gap refers to the lack of information about the long-term duration of immunogenicity and effectiveness of YF fractional vaccination as compared to the full dose (13).

Currently, there are a few world producers of YF vaccine prequalified by the World Health Organization (WHO) and the increasing demand for YF vaccine has become a challenge and impacted the international YF vaccine stockpile. Approximately 450 million doses are estimated to achieve high vaccination coverage (above 80%) in areas of YF viral circulation but the annual production of YF vaccine accomplishes only 80 million doses, rendering at risk populations susceptible to the infection (14, 15). Considering the current YF epidemiological scenario worldwide, a set of measures has been proposed by the WHO to improve YF vaccine supply, including the recommendation of a dose sparing strategy, as a short-term measure (16). Roukens et al. (17) have proposed that intradermal administration of one fifth of the amount of YF vaccine results in seropositivity in all volunteers (17). In 2016, the Strategic Advisory Group of Experts (SAGE) from the WHO considered that the available evidences were sufficient to determine the use of fractional dose, as a safe and effective option for mass vaccination campaigns to control urban outbreaks in situations of YF vaccine shortage (11, 12, 17).

Previous studies carried out by the Collaborative Group for Studies of Yellow Fever Vaccine (FIOCRUZ-Brazil) in 2009, as clinical dose-response cohort investigations with the 17DD-YF vaccine (18, 19) have further contributed to support the use of lower dose of YF vaccine. Together, Martins et al. (18) and Campi-Azevedo et al. (19) demonstrated that doses above 3,013IU elicit similar levels of neutralizing antibodies, equivalent peak viremia and strong pro-inflammatory response in a similar timeline as compared to the reference full dose (27,476IU). Moreover, it was shown that the YF-specific immunity lasted up to 1-year after primary vaccination with satisfactory levels of neutralizing antibodies (18, 19).

The knowledge about the long-term duration of YF-specific immunity after primary vaccination with lower doses is crucial to support and strengthen the use of fractional dose strategy. Recently, Martins et al. (18) have evaluated the status of YF-specific immunity in those participants on the dose-response study in 2009 and demonstrated that seropositivity was maintained in 85% of vaccinees across groups that received subdoses of 17DD-YF vaccine (20). Moreover, Roukens et al. (21) have reported that intradermal administration of a one-fifth dose of 17D-YF vaccine induced a protective immune response that lasted for 10 years after vaccination (21).

The current study is a complementary investigation based on a parallel analysis of humoral and cellular immunity in a subset of volunteers originally enrolled in the dose-response study in 2009. There is no precedent follow-up study that simultaneously evaluate the long-term duration of humoral and cellular immunity upon 17DD-YF vaccination with lower doses. These findings will add new evidences to support the regular use of dose sparing strategy for YF vaccine in adults.

## Materials and Methods

### Study Population

**Figure 1:**
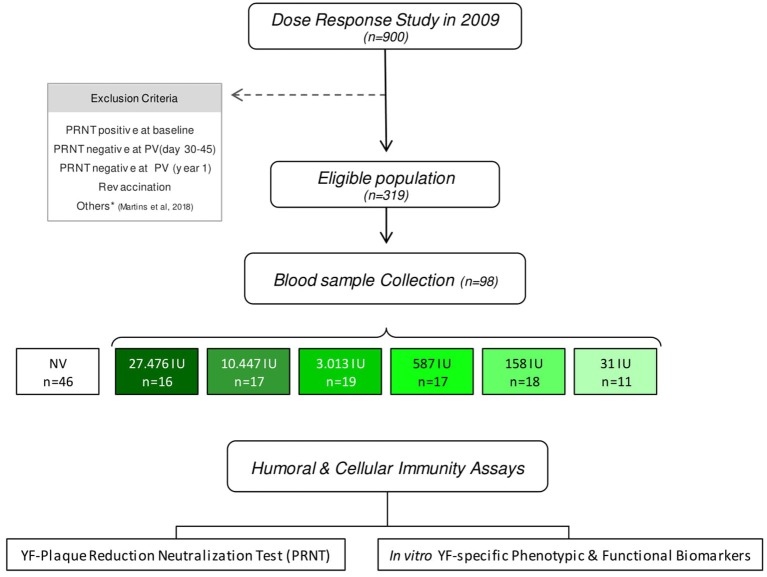
Study design flowchart. The consort diagram summarizes the study steps. This is a 8-years follow-up study, of adult male army conscripts from Rio de Janeiro, average age of 19.4 years old, who had received the reference full dose and subdoses of 17DD-YF vaccine during the dose-response study in 2009 (12). From the eligible population (*n* = 319), only 98 volunteers agreed to provide two blood samples, one without anticoagulant for humoral analysis and an additional heparinized sample for cellular immunity assays. These volunteers were categorized into six groups, according to the dose of 17DD-YF vaccine administered in 2009: 27,476IU, considered the reference dose (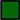, *n* = 16); 10,447IU (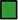, *n* = 17); 3,013IU (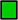, *n* = 19); 587IU (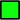, *n* = 17); 158IU (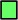, *n* = 18), and 31IU (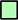, *n* = 11). An additional group of non-vaccinated adult male army conscripts, referred as NV(day0), was included as a control (□, *n* = 46). Humoral and cellular immunity profile was determined for each volunteer using the YF-plaque reduction neutralization test (PRNT) and *in vitro* YF-specific phenotypic & functional biomarkers.

The present study was designed by the Collaborative Group for Studies of Yellow Fever Vaccine as an extension of the dose-response study with the 17DD YF-vaccine conducted by Bio-Manguinhos in 2009, to evaluate the YF-specific humoral and cellular immunity duration and to provide supportive evidences for the use of fractional doses. The original dose-response study carried out in 2009, when the volunteers were selected was a non-inferiority, double blind, randomized clinical trial of immunogenicity and safety. The current investigation is a 8-years follow-up investigation that enrolled adult male army conscripts from Rio de Janeiro (a non-endemic area for YF in 2009), average age of 19.4 years old, who had received the reference full dose and subdoses of 17DD-YF vaccine during the dose-response study in 2009 (18).The target subjects were those with negative PRNT levels before vaccination in 2009 and who were not revaccinated. A total of 319 volunteers adhered to the current protocol and were eligible for the study. Exclusion criteria followed those described by Martins et al. (18), de Menezes Martins et al. (20) and briefly included: PRNT seropositivity at baseline in 2009, PRNT seronegativity at 30–45 days or 1-year upon primary vaccination and YF re-vaccination. Participants that had participated on military missions or traveled to sylvatic areas of YF viral circulation after 2009 were also excluded from the current investigation. Blood sampling was performed by qualified nursing team at FIOCRUZ, following informed consent and no access to additional clinical records was planned in the original study protocol. Before blood collection, participants were asked at least twice if they had been revaccinated. They were also asked to confirm that they had participated in the dose-response study in 2009. From the eligible population (*n* = 319), 98 volunteers agreed to participate in this study and were categorized into six groups, according to the dose of 17DD-YF vaccine administered in 2009: 27,476IU, considered as the reference full dose (*n* = 16); 10,447IU (*n* = 17); 3,013IU (*n* = 19); 587IU (*n* = 17); 158IU (*n* = 18) and 31IU (*n* = 11). In the original study, a single dose of 0.5 mL of 17DD-YF vaccine formulations with decreasing amounts of viral particles was given to each participant. An additional group of 46 adult male army conscripts from a database of another study carried out by our own group (22) was included as non-vaccinated controls and referred as NV (day0). Detailed compendium of the study population and methods are provided in the study design flowchart showed in the Figure 1.

Whole blood samples were collected from each volunteer, including 5 mL in tubes without anticoagulant for YF-plaque reduction neutralization test (PRNT) and 20 mL in heparin sodium for 17DD-YF phenotypic and functional analyses of cellular immunity profile.

This is an 8-years follow-up study included in a clinical trial registry (NCT 03338231). The study protocol was approved by the Ethics Committee at Instituto Nacional de Infectologia Evandro Chagas, FIOCRUZ (Plataforma Brasil, CAAE#65823617.6.3001.5091). All procedures followed the Helsinki Declaration, the Brazilian ethical standards of scientific research involving human subjects and the good clinical practices.

### Serology for YF-Plaque Reduction Neutralizing Test (PRNT)

The PRNT levels to the 17DD-YF virus were quantified using the same method employed in the dose-response study of 2009 (18), using the same cut-off for seropositivity: >2.7 log_10_mIU/mL (501.2 mIU/mL), or about 1/20 in dilution. The PRNT analysis was performed at Laboratório de Tecnologia Virológica, Bio-Manguinhos (LATEV, FIOCRUZ-RJ, Brazil).

### *In vitro* Assays for YF-Specific Phenotypic and Functional Memory Biomarkers

The peripheral blood lymphoproliferation assay for measuring YF-specific cellular immunity memory was performed as previously described by Costa-Pereira et al. (22). Briefly, aliquots (1.0 × 10^6^/well) of peripheral blood mononuclear cells (PBMC) were incubated in the absence (Control) or presence of 17DD-YF vaccine stimuli (17DD-YF Ag), at 37°C in a 5% CO_2_ for 6 days. After incubation, PBMC were harvested and stained with live/dead dye (Life Technologies, Carlsbad, CA, USA) and a mix of monoclonal antibodies (mAbs) [anti-CD4/(RPA-T4)/FITC; anti-CD8/(SK1)/PerCP-Cy5.5; anti-CD27/(M-T271)/PE, anti-CD45RO/(UCHL1)/PE-Cy7 and anti-CD3/(SK7)/APC-Cy7] to quantify memory T-cell subsets and [anti-CD19/(HIB19)/PerCP, anti-CD27/(M-T271)/PE and anti-IgD/(IA6-2)/FITC] for B-cell analysis. All mAbs were purchased from BD Pharmingen (BD Bioscience, San Diego, CA, USA).

Additional aliquots of cultured PBMC were labeled with live/dead dye and a cocktail of mAbs [anti-CD3/(UCHT1)/Qdot605 (Invitrogen, Carlsbad, CA, USA); anti-CD4/(GK1.5)/APCe-Fluor780 (eBioscience, San Diego, CA, USA); anti-CD8/(SK1)/PerCP (BD Biosciences, San Diego, CA, USA) and anti-CD19/(HIB19)/Alexa-Fluor700 (eBioscience, San Diego, CA, USA)] to identify T-cell subsets and B-cells. Following the Fix/Perm step, cells were reincubated with a mix of mAbs [anti-TNF-α/(clone MAb11)/PE-Cy7; anti-IFN-γ/(clone B27)/Alexa-Fluor488); anti-IL-5/(JES1-39D10)/PE and anti-IL-10/(JES3-19F1)/APC, all purchased from BD Bioscience] to enumerate functional status of T and B-cells.

Stained PBMC were fixed and stored at 4°C up to 24 h prior to flow cytometric acquisition on a BD LSR Fortessa (BD Bioscience, San Diego, CA, USA).

A total of 100,000 events were acquired per each sample and data stored for offline analysis. The FlowJo software (version 9.3.2, TreeStar, San Diego, CA, USA) was employed for data analysis. The expression of CD45RO and CD27 was used to define memory CD4^+^ and CD8^+^ T-cell subsets: Naïve/NCD4;NCD8 – (CD27^+^CD45RO^−^); early Effector/eEfCD4;eEfCD8 – (CD27^−^CD45RO^−^); Central Memory/CMCD4;CMCD8 – (CD27^+^CD45RO^+^) and Effector Memory/EMCD4;EMCD8 – (CD27^−^CD45RO^+^). The expression of IgD and CD27 was employed to define memory B-cell subsets: Naïve/NCD19 – (CD27^−^IgD^+^); Non-classical Memory/nCMCD19 – (CD27^+^IgD^+^) and Classical Memory/CMCD19 – (CD27^+^IgD^−^). Functional CD4^+^ and CD8^+^ T-cell subsets (TNF-α, IFN-γ, IL-10, and IL-5) as well as B-cells (TNF-α, IL-10, and IL-5) were also quantified. The results were first generated as percentage of memory T and B-cell subsets in parallel with cytokine^+^-cells. The final results of YF-specific phenotypic and functional memory biomarkers were presented as 17DD-YF Ag/Control culture Index, calculated as the ratio between the percentages of cells observed in the 17DD-YF Ag cultures by percentage observed in the respective control culture.

### Statistical Analysis

Statistical analysis was performed, first blindly, by using ANOVA adjusted to multiple comparisons and Fisher's exact test, as indicated. After code unblinding, each 17DD-YF vaccine group was compared to the control group of non-vaccinated subjects –NV(day0). The iterative proportion fitting of PRNT seropositivity rates were also calculated for comparative analysis amongst groups. In all cases, significant differences were considered at *p* < 0.05 and highlighted by asterisk (^*^) as compared to NV(day0).

## Results

### Similar Immunogenicity Is Maintained 8-Years After 17DD-YF Primary Vaccination With Lower Doses

**Figure 2:**
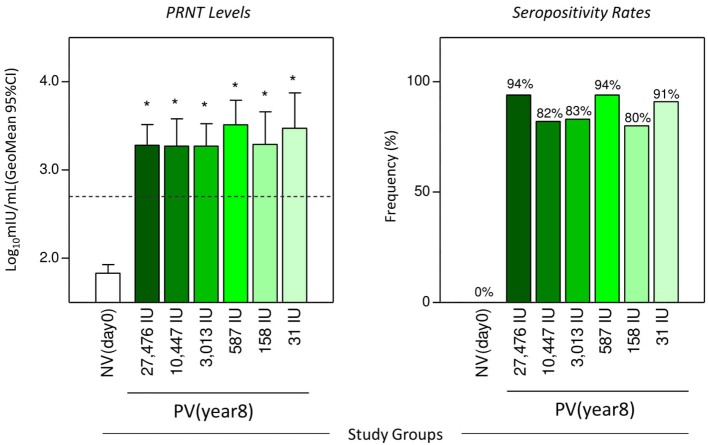
Neutralizing antibodies levels and PRNT seropositivity rates 8-years after 17DD-YF primary vaccination with different doses. The YF-specific plaque reduction neutralizing antibodies were measured using the same method employed in the dose-response study of 2009 (12). The cut-off for seropositivity was set at > 2.7 log_10_ mIU/mL (501.2 mIU/mL) or about 1/20 in dilution. The eligible vaccinees (*n* = 98) were categorized into six subgroups, according to the dose of 17DD-YF vaccine administered in 2009: 27,476IU, considered the reference dose (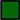, *n* = 16); 10,447IU (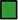, *n* = 17); 3,013IU (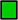, *n* = 18); 587IU (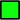, *n* = 17); 158IU (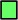, *n* = 15), and 31IU (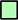, *n* = 11). A group of non-vaccinated adult male army conscripts, referred as NV(day0), was included as a control (□, *n* = 46). The PRNT levels were expressed as geometric mean titer and 95%CI of log_10_ mIU/mL and the seropositivity rates shown as frequency (%) of subjects above the cut-off edge (2.7 log_10_ mIU/mL—dashed line). Comparative analysis of PRNT levels and seropositivity rates were assessed by ANOVA adjusted to multiple comparisons and Fisher exact test, respectively. In all cases, significant differences were considered at *p* < 0.05 and highlighted by asterisk (^*^) as compared to NV(day0).

The geometric mean titers of YF-specific plaque reduction neutralizing antibodies and the frequency of seropositive vaccinees (>2.7 log_10_ mIU/mL) at 8-years upon 17DD-YF primary vaccination with different doses are presented in the Figure 2. The 8-years follow-up study demonstrated that volunteers, who had seroconverted upon 17DD-YF primary vaccination with lower doses and had not been revaccinated, still presented similar antibody levels and seropositivity rates as in all subdoses administrated as compared to the reference full dose (Figure 2).

Aiming to determine the overall seropositivity rates achieved by the primary vaccination with lower doses of 17DD-YF vaccine, since the seroconversion at 30–45 days throughout the 8-years time span follow-up, an iterative proportion fitting of PRNT seropositivity rates was calculated as a progressive decrease on seropositivity rates along time and data presented in the Supplementary Figure 1. The results demonstrated that at 30–45 days, doses from 27,476IU (reference full dose) above 587IU displayed similar immunogenicity (96, 100, 97, and 97%, in that order), while doses of 158IU and 31IU induced lower PRNT levels (89 and 56%, respectively). The 1-year follow-up included only volunteers who had seroconverted at day 30–45 after vaccination and had not been re-vaccinated. Seropositivity was maintained up to 1-year across doses (99, 97, 99, 100, and 97%, correspondingly), except for the 31IU group that lead to 89% of seropositivity. The present study, carried out 8 years after primary vaccination, included only volunteers who had seroconverted at day 30–45 after vaccination, maintained the seropositivity at 1-year and had not been revaccinated. Subjects with negative PRNT at 30–45 days or 1-year after primary vaccination were not included in the present investigation since they were re-vaccinated as part of the study protocol approved in 2009. The results demonstrated an overall PRNT seropositivity rate of 87%, with similar rates across groups (94, 82, 83, 94, 80, and 91%, correspondingly). However, the iterative proportion fitting of seropositivity rates demonstrated that only doses above 587IU elicited similar immunogenicity (89, 80, 80, and 91%, respectively), whereas doses of 158IU and 31IU reached lower proportion of seropositivity rates (68 and 46%, in that order) (Supplementary Figure 1).

### Equivalent YF-Specific Cellular Memory Is Observed 8-Years After 17DD-YF Primary Vaccination With Lower Doses

**Figure 3:**
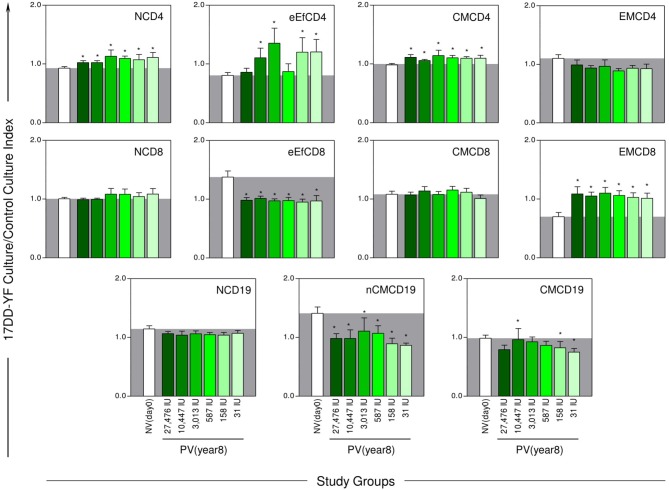
YF-specific phenotypic memory biomarkers 8-years after 17DD-YF primary vaccination with different doses. The YF-specific phenotypic memory was measured based on cell-surface marker expression upon *in vitro* 17DD-YF antigen recall as described by Costa-Pereira et al. (22). Flow cytometric phenotypic analysis based on the expression of CD45RO and CD27 was used to define memory CD4^+^ and CD8^+^ T-cell subsets: Naïve/NCD4;NCD8—(CD27^+^CD45RO^−^); early Effector/eEfCD4;eEfCD8—(CD27^−^CD45RO^−^); Central Memory/CMCD4;CMCD8—(CD27^+^CD45RO^+^) and Effector Memory/EMCD4;EMCD8—(CD27^−^CD45RO^+^). The expression of IgD and CD27 was employed to define memory B-cell subsets: Naïve/NCD19—(CD27^−^IgD^+^); Non-classical Memory/nCMCD19—(CD27^+^IgD^+^) and Classical Memory/CMCD19—(CD27^+^IgD^−^). The eligible vaccinees (*n* = 98) were categorized into six subgroups, according to the dose of 17DD-YF vaccine administered in 2009: 27,476IU, considered the reference dose (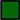, *n* = 16); 10,447IU (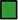, *n* = 17); 3,013IU (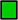, *n* = 19); 587IU (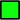, *n* = 17); 158IU (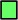, *n* = 18), and 31IU (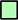, *n* = 11). A group of non-vaccinated adult male army conscripts, referred as NV(day0), was included as a control (□, *n* = 46). The results are expressed mean values and standard error of 17DD-YF Ag/CC Index as described in Material and Methods. Comparative analysis for each biomarker amongst groups were assessed by ANOVA adjusted to multiple comparisons and significant differences at *p* < 0.05 highlighted by asterisk (^*^) as compared to the mean values observed for NV(day0). The gray zone represents the mean values observed for non-vaccinated controls.

**Figure 4:**
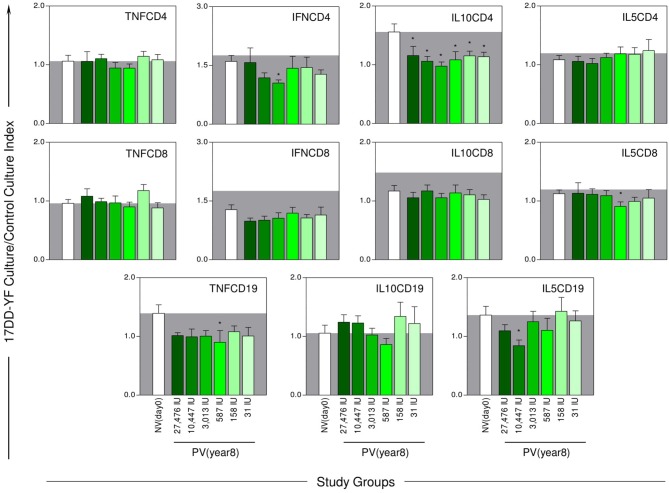
YF-specific functional memory biomarkers 8-years after 17DD-YF primary vaccination with different doses. The YF-specific functional memory was measured as intracytoplasmic cytokine profile upon *in vitro* 17DD-YF antigen recall as described by Costa-Pereira et al. (22). Flow cytometric staining were performed to quantity functional CD4^+^ and CD8^+^ T-cell subsets producing TNF-α, IFN-γ, IL-10, and IL-5 as well as B-cells producing TNF-α, IL-10, and IL-5. The eligible vaccinees (*n* = 98) were categorized into six groups, according to the dose of 17DD-YF vaccine administered in 2009: 27,476IU, considered the reference dose (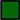, *n* = 16); 10,447IU (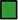, *n* = 17); 3,013IU (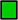, *n* = 19); 587IU (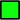, *n* = 17); 158IU (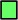, *n* = 18), and 31IU (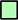, *n* = 11). A group of non-vaccinated adult male army conscripts, referred as NV(day0), was included as a control (□, *n* = 4 6). The results are expressed mean values and standard error of 17DD-YF Ag/CC Index as described in Material and Methods. Comparative analysis for each biomarker amongst groups were assessed by ANOVA adjusted to multiple comparisons and significant differences at *p* < 0.05 highlighted by asterisk (^*^) as compared to the mean values observed for NV(day0). The gray zone represents the mean values observed for non-vaccinated controls.

The YF-specific phenotypic and functional memory profiles at 8-years after 17DD-YF primary vaccination with different doses are shown in the Figures 3, 4. The YF-specific memory biomarkers were assessed upon *in vitro* 17DD-YF antigen recall and the results presented as 17DD-YF Ag/Control culture Index.

Analysis of phenotypic memory biomarkers revealed, regardless of the 17DD-YF dose used at primary vaccination, higher levels of NCD4 in all vaccinees as compared to the NV(day0) control group. Conversely, lower levels of eEfCD8 and nCMCD19 were similarly observed across groups as compared to the NV(day0) control group. Waves of eEfCD4 were observed in 4 out of 5 lower doses tested (10,447IU, 3,013IU, 158IU, and 31IU). Three particular profiles were observed for CMCD19, including higher levels in 10,447IU and lower levels in 158IU and 31IU groups. Of note were the increased levels of CMCD4 and the remarkable levels of EMCD8 observed in all vaccinees even 8-years upon 17DD-YF primary vaccination with lower doses (Figure 3).

The data from the current investigation was compared with those from another study carried out by our own group, in which the participants received the “routine” full dose of 17DD-YF vaccine (13). In both studies the results were obtained using the same methods to quantify the levels of neutralizing antibodies and EMCD8. Using the combined database from both studies, the YF-specific PRNT and EMCD8 profiles were compared and the results presented in the Supplementary Figure 2. Data analysis demonstrated that 8-years after primary vaccination with17DD-YF subdoses, all selected vaccinees still presented preserved levels of PRNT and EMCD8, similar to those observed in PV(day30–45) and PV(year7–9) but higher than that observed in NV(day0) and PV(year>10).

The analysis of functional biomarkers demonstrated equivalent lower levels of IL10CD4 across groups. Several random point profiles, such as lower levels of IL5CD19, IFNCD4 along with TNFCD19 and IL5CD8, were observed in 10,447IU, 3,013IU, and 587IU groups, respectively (Figure 4).

Together, these findings demonstrated that volunteers, who had seroconverted upon 17DD-YF primary vaccination with lower doses and had not been revaccinated, still presented in an 8-years follow-up study comparable YF-specific cellular memory, particularly CMCD4 and EMCD8 as compared to the reference full dose (Figure 3).

### Comparable Biomarker Network Portrait Is Perceived 8-Years After 17DD-YF Primary Vaccination With Different Doses

**Figure 5:**
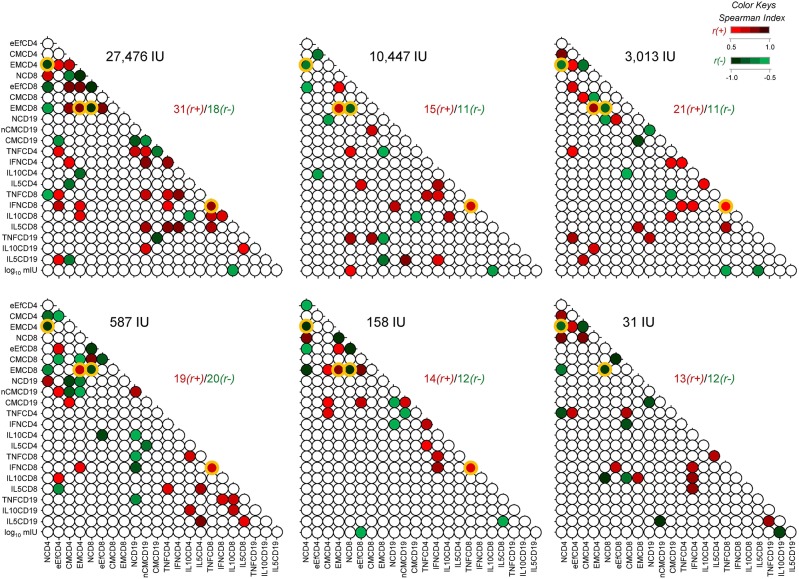
Biomarker network matrices 8-years after 17DD-YF primary vaccination with different doses. The biomarker network of YF-specific humoral and cellular memory was built to define the connections between PRNT levels (log_10_ mIU/mL), phenotypic (NCD4, eEfCD4, CMCD4, EMCD4, NCD8, eEfCD8, CMCD8, EMCD8, NCD19, nCMCD19, and CMCD19) and functional memory attributes (TNFCD4, IFNCD4, IL10CD4, IL5CD4, TNFCD8, IFNCD8, IL10CD8, IL5CD8, TNFCD19, IL10CD19, and IL5CD19). Correlation analysis were carried out for six vaccinees groups, according to the dose of 17DD-YF vaccine administered in 2009: 27,476IU, considered the reference dose; 10,447IU; 3,013IU; 587IU; 158IU, and 31IU. Matrices were assembled in dotted template with each dot representing a correlation axis between two attributes. Color keys were employed to identify significant Spearman's correlation “r” indices at *p* < 0.05, referred as positive (red scale, 
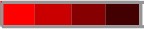
, r(+) ranging from 0.5 to 1.0) or negative (green scale, 
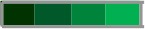
, r(-) ranging from −1.0 to −0.5). Non-significant correlations are represented by white dots. Ratio between positive and negative correlations “r(+)/r(-)” are provided in the Figure. The common correlations across distinct 17DD-YF vaccine doses are highlighted by orange frames.

Biomarker networks were built to define connections between YF-specific humoral and cellular memory at 8-years follow 17DD-YF primary vaccination with different doses (Figure 5). In general, there was a predominance of positive correlations “*r(*+*)*/*r(-)*” between YF-specific humoral and cellular memory at 8-years upon primary vaccination in most subgroups, with higher ratios for doses of 27,476IU, 10,447IU, and 3,013IU (31/18, 15/11, and 21/11, respectively). Strong negative correlations were observed for “EMCD4,1/NCD4” and “EMCD8,1/NCD8” in all groups, regardless of the dose administrated. Noteworthy was the strong positive correlation observed for “EMCD4,EMCD8” and “TNFCD8,IFNCD8” in most groups, except for volunteers receiving the 31IU dose (Figure 5).

### Analogous Snapshot Highlights the Major Similarities of YF-Specific Humoral and Cellular Memory Profile 8-Years After 17DD-YF Primary Vaccination With Different Doses

**Figure 6:**
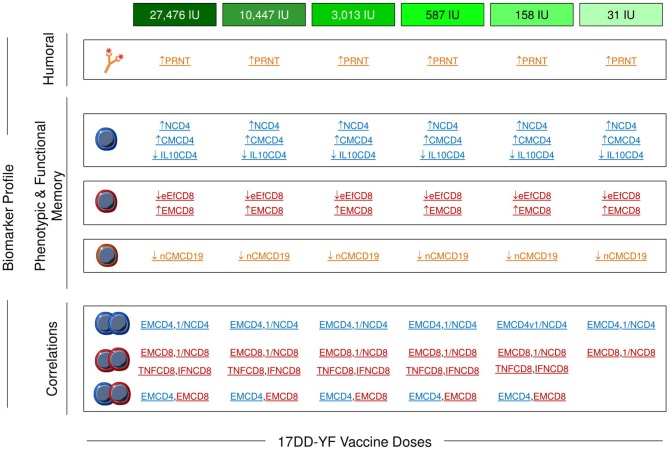
Snapshot overview of major similarities of YF-specific humoral and cellular memory profile 8-years after 17DD-YF primary vaccination with different doses. Selection of biomarkers with similar profile throughout distinct subdoses of 17DD-YF vaccine (27,476IU - reference dose; 10,447IU; 3,013IU; 587IU; 158IU and 31IU). Humoral memory immunity (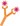 = PRNT) was measured by YF-specific plaque reduction neutralizing antibodies using the same method employed in the dose-response study of 2009 (9). The YF-specific phenotypic and functional memory biomarkers (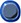 = NCD4, CMCD4, and IL10CD4; 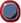 = eEfCD8 and EMCD8 along with 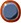 = nCMCD19) were measured upon *in vitro* 17DD-YF antigen recall as described by Costa-Pereira et al. (22). Arrows highlighted decreased (↓) or increased levels (↑) as compared to NV(day0). Correlation analysis were carried out to identify significant negative (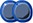 = “EMCD4,1/NCD4” and 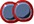 = “EMCD8,1/NCD8”) or positive (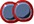 = “TNFCD8,IFNCD8” and 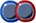 = “EMCD4,EMCD8”) common correlations throughout distinct subdoses of 17DD-YF vaccine (27,476IU - reference dose; 10,447IU; 3,013IU; 587IU; 158IU and 31IU), except the correlations “TNFCD8,IFNCD8” and “EMCD4,EMCD8” not observed for the lower dose (31IU).

The selection of biomarkers presenting common profiles throughout distinct doses of 17DD-YF vaccine is compiled in the Figure 6. Humoral immunogenicity, phenotypic and functional cellular memory and correlation links between attributes were assembled to identify universal biomarkers across the groups. Arrows were employed to highlight decreased (↓) or increased levels (↑) as compared to NV(day0). Data analysis revealed that a range of attributes (↑PRNT, ↑NCD4, ↑CMCD4, ↓IL10CD4, ↓eEfCD8, ↑EMCD8, ↓nCMCD19) presented similar profiles throughout the distinct 17DD-YF vaccine doses even 8-years after primary vaccination (Figure 6). Moreover, negative correlations between “EMCD4,1/NCD4” and “EMCD8,1/NCD8” along with positive correlation for “EMCD4,EMCD8” and “TNFCD8,IFNCD8” were also observed. Worth mentioning was the preserved PRNT titers, the gold standard to measure post-vaccination immunity to YF, and the outstanding levels of EMCD8, a relevant correlate of protection for YF-specific cellular immunity (Figure 6).

## Discussion

The current study is a complementary investigation that simultaneously evaluate the long-term duration of humoral and cellular immunity in a subset of volunteers originally receiving subdoses of 17DD-YF vaccine, enrolled in the dose-response study in 2009.

Few studies have addressed the duration of immunity after immunization with reduced doses of YF vaccine. The fractional dose of 17DD-YF vaccine has been shown to be safe and non-inferior to the standard dose in inducing seroprotection (17–19). The seropositivity rates reported by Ahuka-Mundeke et al. (23) in a recent vaccination campaign using one-fifth fractional-dose in Africa are similar to those obtained by Martins et al. (18) and Campi-Azevedo et al. (19), showing 98% seroconversion at 30-45 days after primary vaccination in seronegative subjects (23). A long-term follow-up randomized, controlled, non-inferiority trial study conducted with the 17D-YF vaccine has shown that a one-fifth dose YF vaccine induced a protective immunity that lasted for 10 years after vaccination (21).

Recently, Martins et al. (18) have evaluated the duration of immunity by measuring neutralizing antibody levels 8-years after the dose-response study conducted in 2009 using subdoses of 17DD-YF vaccine. Seropositivity was maintained in 85% of 318 participants and was similar across groups as compared to the reference full dose. The present results demonstrated that, after 8-years, subjects who had seroconverted after 17DD-YF primary vaccination still presented an overall seropositivty rate of 87%, similar to that observed by Martins et al. (18). Vaccinees who received doses from 10.447IU down to 31IU still presented high seropositivity rates (82, 83, 94, 80, and 91%, in that order) comparable to that reported for the reference full dose (94%). It is important to mention that only volunteers who had seroconverted at 30–45 days after primary vaccination in 2009 and maintained the seropositivity at 1-year with no records of revaccination have been included in the present investigation. However, in order to overcome this putative drawback, the overall seropositivity has been calculated as iterative proportion fitting of PRNT seropositivity rates since the seroconversion at 30-45 days throughout the 8-years time span follow-up. The data demonstrated that doses higher then 587IU displayed similarly high immunogenicity while doses of 158IU and 31IU induced lower PRNT levels. Which was a direct reflection of the higher failure of primary seroconversion at 30–45 days observed for the two lowest doses (158IU and 31IU) (18, 19). Therefore, these doses should be considered inferior to the other doses tested.

The results also pointed out that biological markers of memory for cellular immunity, particularly EMCD8, were found in comparable levels across groups. Noteworthy was the strong positive correlations (“EMCD4,EMCD8” and “TNFCD8,IFNCD8”) observed in most subdoses, except for 31IU. A predominance of positive correlations between YF-specific humoral and cellular memory was observed in most groups at 8-years upon primary vaccination, with higher ratios found for doses 27,476IU, 10,447IU, and 3,013IU. Moreover, the high number of connectivity triggered by the reference full vaccine dose (27,476IU) may represent the massive activation of a wide range of cell clones elicited by the high antigenic concentration. Although the number of connectivity drops significantly in lower YF vaccine doses, the correlates of protection (PRNT and EMCD8) still remains detectable in similar levels across all doses. These findings allude to those previously reported by Campi-Azevedo et al. (19) that higher similarities in immunological and virological parameters were found for subdoses down to 3,013IU as compared to the reference full dose (27,476IU), while lower subdoses elicited an impaired magnitude of equivalence. Therefore, the biomarkers associated the preserved connections such as “EMCD4,EMCD8”, “TNFCD8,IFNCD8” besides “EMCD4,1/NCD4” and “EMCD8,1/NCD8” might represent relevant axis to guarantee the protective immunity upon 17DD-YF vaccination.

Major similarities underscored the preserved PRNT titers and the outstanding levels of EMCD8, relevant correlates of protection for YF-specific immunity. The neutralizing antibodies levels have been considered the convenient proxy correlate of protection to monitor the immunogenicity of YF vaccination (3). Several studies have demonstrated that neutralizing antibodies and YF-specific CD8^+^ T-cell are relevant imprints of immunological memory induced by YF vaccination (22, 24–28). The protective immune memory developed after YF vaccination comprises the ability to produce YF-neutralizing antibodies together with the generation of effector memory CD8^+^ T-cell. The high magnitude, broad specificity, robust proliferative profile, multiple functions and long-term persistence of immune memory mediated by CD8^+^ T-cells is an attribute that defines efficacious immune response after vaccination (29). Focusing on these two premium biomarkers (PRNT and EMCD8), an additional analysis was performed using a combined database from the current investigation and from another study of our own group using the “routine” full dose of 17DD-YF vaccine (22). The results further supports that 8-years after primary vaccination with17DD-YF subdoses all selected vaccinees still presented preserved levels of correlates of protection.

Together, the current findings demonstrated that the duration of immunity upon vaccination of adults with subdoses of 17DD-YF vaccine is acceptable and comparable with that observed for the reference full dose, except for 158IU and 31IU groups that elicited lower iterative proportion of PRNT seropositivity rates along time. The PRNT levels and seropositivity rates along with the profile of EMCD8 provide relevant evidences to support the use of dose sparing strategy for YF vaccine in adults.

Dose-response studies are still required for its universal use in children <2-years of age, elderly, pregnant women as well as immunocompromised patients considering the particularities of their immune response. Of our knowledge, recent studies are currently under investigation in Africa to evaluate fractional dose YF vaccination in children, adults and HIV-infected subjects. A dose-response study in children has been considered by our group and has being submitted for evaluation. The limited data on duration of protection also does not qualify people for international travel under the International Health to receive fractional dose vaccination.

## Data Availability

All datasets generated for this study are included in the manuscript and/or the Supplementary Files.

## Ethics Statement

This study was carried out in accordance with the recommendations of Brazilian ethical standards of scientific research involving humans and the good clinical practices with written informed consent from all subjects. All subjects gave written informed consent in accordance with the Declaration of Helsinki. The protocol was approved by the Ethics Committee at Instituto Nacional de Infectologia Evandro Chagas, FIOCRUZ (Plataforma Brasil, CAAE#65823617.6.3001.5091).

## Author Contributions

AC-A, MM, LC, RF, RM, AH, AR, CD, AT-C, and OM-F: designing research study. RM, OM-F, AR, and CD: funding acquisition. IC-R, AC-A, VP-M, JC-d-R, JF, TS-L, LR, LF, CC-P, SL, AT-C, and OM-F: conducting experiments. MM, TN, JX, EA, RF, and TC: field study. AC-A, VP-M, JC-d-R, JF, and SL: acquiring data. IC-R, AC-A, LF, JM, AT-C, and OM-F: analyzing data. TN, JX, EA and TC: validation. RM and AH: advisory committee. IC-R, AC-A, JC-d-R, CC-P, LR, JM, AT-C and OM-F: writing the manuscript.

### Conflict of Interest Statement

The authors declare that the research was conducted in the absence of any commercial or financial relationships that could be construed as a potential conflict of interest.
