# Corrigendum: Duration of Humoral and Cellular Immunity 8 Years After Administration of Reduced Doses of the 17DD-Yellow Fever Vaccine

**DOI:** 10.3389/fimmu.2019.02433

**Published:** 2019-10-22

**Authors:** Ismael Artur da Costa-Rocha, Ana Carolina Campi-Azevedo, Vanessa Peruhype-Magalhães, Jordana Grazziela Coelho-dos-Reis, Jordana Rodrigues Barbosa Fradico, Thalles Souza-Lopes, Laise Rodrigues Reis, Larissa Chaves Freire, Christiane Costa-Pereira, Juliana Vaz de Melo Mambrini, Maria de Lourdes de Sousa Maia, Sheila Maria Barbosa de Lima, Tatiana Guimarães de Noronha, Janaina Reis Xavier, Luiz Antonio Bastos Camacho, Elizabeth Maciel de Albuquerque, Roberto Henrique Guedes Farias, Thalita da Matta de Castro, Akira Homma, Alessandro Pecego Martins Romano, Carla Magda Domingues, Reinaldo de Menezes Martins, Andréa Teixeira-Carvalho, Olindo Assis Martins-Filho

**Affiliations:** ^1^Grupo Integrado de Pesquisas em Biomarcadores, Instituto René Rachou, Fundação Oswaldo Cruz – FIOCRUZ-Minas, Belo Horizonte, Brazil; ^2^Laboratório de Virologia Básica e Aplicada, Departamento de Microbiologia, Instituto de Ciências Biológicas, Universidade Federal de Minas Gerais, Belo Horizonte, Brazil; ^3^Núcleo de Estudos em Saúde Pública e Envelhecimento, Instituto René Rachou, Fundação Oswaldo Cruz – FIOCRUZ-Minas, Belo Horizonte, Brazil; ^4^Assessoria Clínica, Instituto de Tecnologia em Imunobiológicos Bio-Manguinhos – FIOCRUZ, Rio de Janeiro, Brazil; ^5^Laboratório de Tecnologia Virológica, Instituto de Tecnologia em Imunobiológicos Bio-Manguinhos – FIOCRUZ, Rio de Janeiro, Brazil; ^6^Departamento de Epidemiologia e Métodos Quantitativos em Saúde - Escola Nacional de Saúde Pública – FIOCRUZ, Rio de Janeiro, Brazil; ^7^Instituto de Biologia do Exército, Rio de Janeiro, Brazil; ^8^Departamento de Vigilância das Doenças Transmissíveis, Secretaria de Vigilância em Saúde, Ministério da Saúde, Brasília, Brazil

**Keywords:** Yellow Fever, 17DD vaccine, subdoses, neutralizing antibodies, cellular memory

In the original article, there was a mistake in Figure 5 as published. One orange frame erroneously shifted slightly to the right. The corrected Figure 5 appears below.

**Figure 5:**
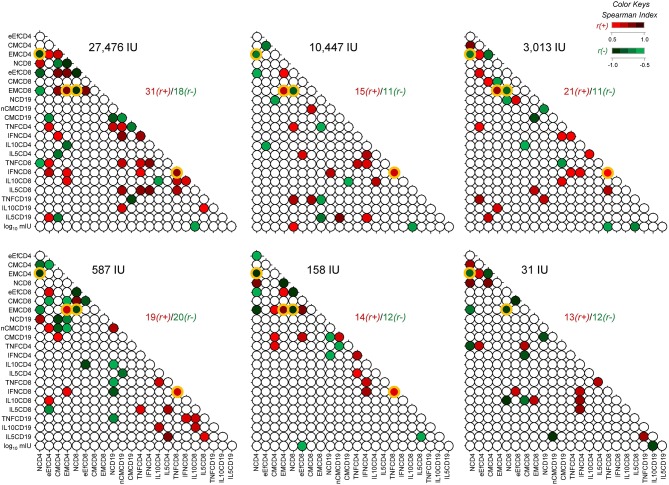
Biomarker network matrices 8-years after 17DD-YF primary vaccination with different doses. The biomarker network of YF-specific humoral and cellular memory was built to define the connections between PRNT levels (log_10_ mIU/mL), phenotypic (NCD4, eEfCD4, CMCD4, EMCD4, NCD8, eEfCD8, CMCD8, EMCD8, NCD19, nCMCD19, and CMCD19) and functional memory attributes (TNFCD4, IFNCD4, IL10CD4, IL5CD4, TNFCD8, IFNCD8, IL10CD8, IL5CD8, TNFCD19, IL10CD19, and IL5CD19). Correlation analysis were carried out for six vaccinees groups, according to the dose of 17DD-YF vaccine administered in 2009: 27,476IU, considered the reference dose; 10,447IU; 3,013IU; 587IU; 158IU, and 31IU. Matrices were assembled in dotted template with each dot representing a correlation axis between two attributes. Color keys were employed to identify significant Spearman's correlation “r” indices at *p* < 0.05, referred as positive (red scale, 
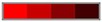
, r(+) ranging from 0.5 to 1.0) or negative (green scale, 
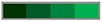
, r(-) ranging from −1.0 to −0.5). Non-significant correlations are represented by white dots. Ratio between positive and negative correlations “r(+)/r(-)” are provided in the Figure. The common correlations across distinct 17DD-YF vaccine doses are highlighted by orange frames.

Additionally, there was a mistake in Supplementary Figure 2 as published. The asterisks indicating statistical significance were erroneously deleted during the JPEG conversion. It is important to mention that no results have been modified. The corrected Supplementary Figure 2 appears below.

**Supplementary Figure 2:**
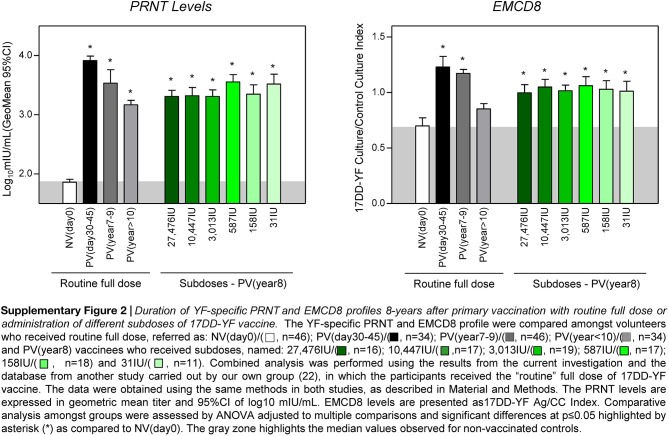


The authors apologize for these errors and state that they do not change the scientific conclusions of the article in any way. The original article has been updated.

