# Supplementary figures and images for: Duration of Humoral and Cellular Immunity 8 Years After Administration of Reduced Doses of the 17DD-Yellow Fever Vaccine
Source: Front Immunol. 2019 Jun 21;10:1211. doi: 10.3389/fimmu.2019.01211 (PMC6598206; doi:10.3389/fimmu.2019.01211)

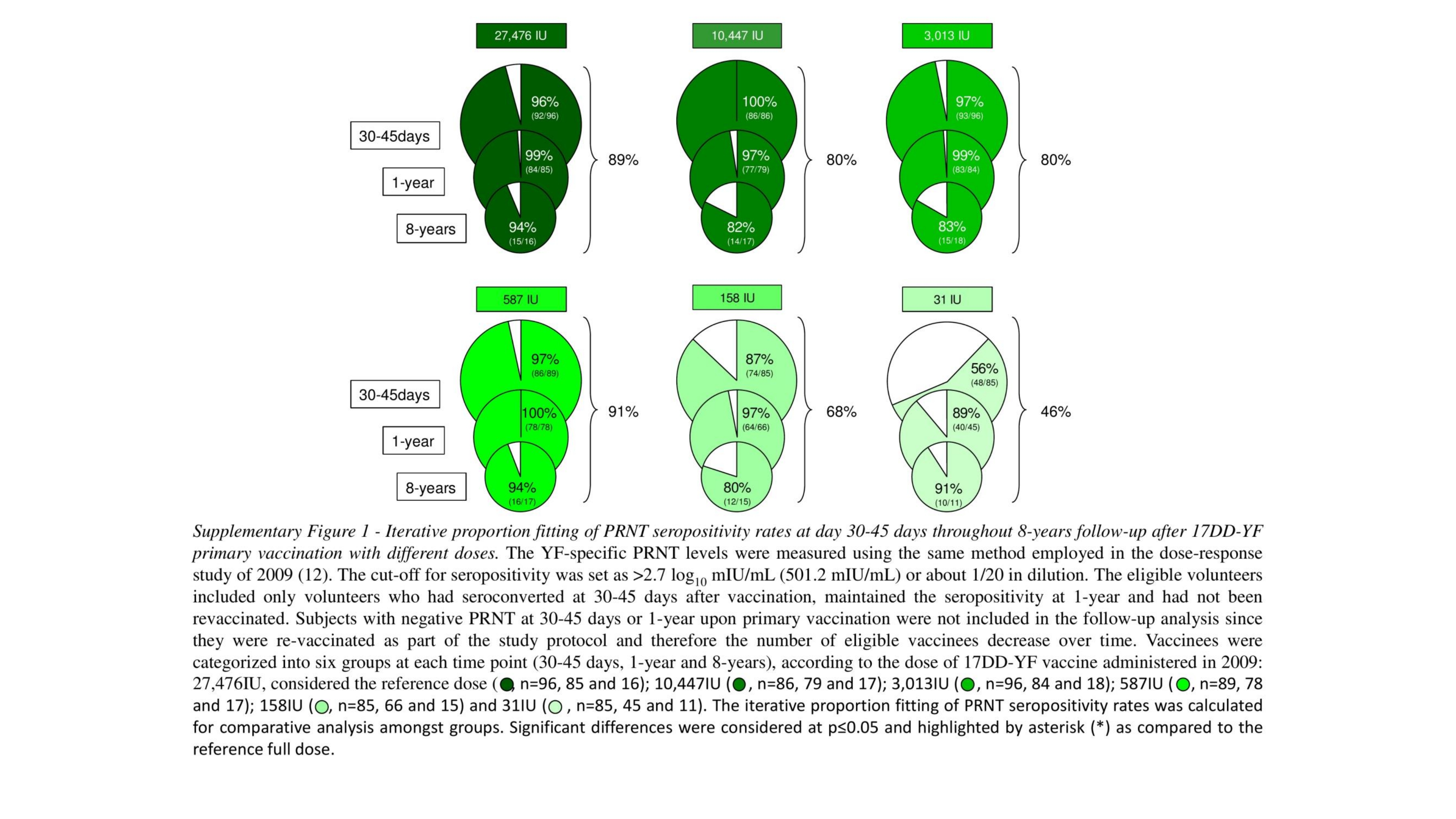

Supplement: Supplementary file 1 [file Image_1.jpg]

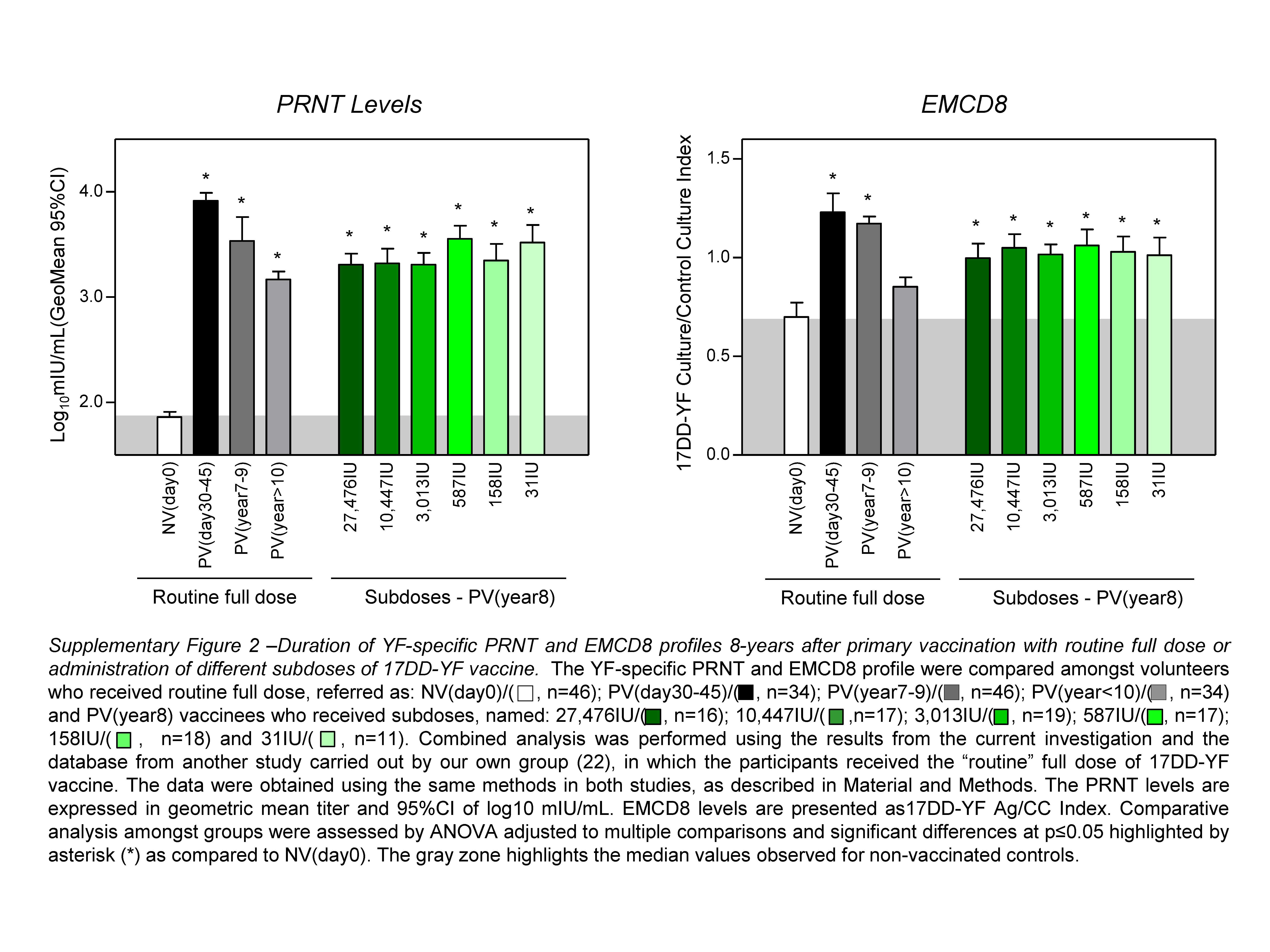

Supplement: Supplementary file 2 [file Image_2.jpg]
